# Combined Immune Therapy Proves Effective in an Advanced Hepatocellular Carcinoma Patient With Poor Liver Reserve: A Case Report

**DOI:** 10.7759/cureus.66164

**Published:** 2024-08-05

**Authors:** Hisashi Nagai, Hao Chen, Ryusuke Karube, Yusuke Koitabashi, Ouka Numata, Kenichi Yamahara

**Affiliations:** 1 Human and Environmental Studies, Tokai University, Kanagawa, JPN; 2 Oncology, Ginza Phoenix Clinic, Tokyo, JPN; 3 Respiratory Medicine, Yokohama City University Hospital, Yokohama, JPN; 4 Regenerative Medicine, Ginza Phoenix Clinic, Tokyo, JPN; 5 Laboratory of Molecular and Cellular Therapy, Institute for Advanced Medical Sciences, Hyogo Medical University, Hyogo, JPN

**Keywords:** α-galactosylceramide, wt1, natural killer cell, dendritic cell, hepatocellular carcinoma, combined immune therapy

## Abstract

There are no effective treatment options for patients with poor performance status and limited liver reserve, classified as Child-Pugh Grade B and C. A 61-year-old man with a prior medical history of hepatitis C virus infection was admitted to the hospital with abdominal distension and significant abdominal ascites. He was diagnosed with stage IVB hepatocellular carcinoma (HCC), characterized by multiple metastases to lymph nodes, lungs, and bones. After receiving combined immune therapy, including dendritic cell therapy targeting WT1 and α-Galactosylceramide, natural killer cells, and Nivolumab, the patient showed significant improvement in HCC and liver reserve function and followed standard treatment. Combined immune therapy is potentially an important option for patients with advanced hepatocellular carcinoma and poor liver reserve function, especially for relatively young patients.

## Introduction

The landscape of cancer immunotherapy is constantly evolving with innovative treatments that harness the body's immune system more effectively to combat malignancy. Among these treatments, integrating dendritic cell (DC) vaccines, natural killer (NK) cell therapy, and immune checkpoint inhibitors such as nivolumab presents a promising trifecta of modalities to enhance anti-tumor immune responses [1]. DC vaccines play a central role in inducing a targeted immune response by presenting tumor-specific antigens to T cells, thereby orchestrating an adaptive immune response tailored to the individual's cancer [2]. Wilms' Tumor 1 (WT1) is commonly used to improve the immune profile, potentially contributing to chemotherapy’s long-lasting and sustained effects [3]. Additionally, the usage of α-Galactosylceramide (α-Galcer) will potentially activate the function of natural killer T (NKT) cells [4].

NK and NKT cells complement this by providing a rapid, innate response capable of destroying tumor cells directly through cytotoxic activity [5]. Their function is crucial in targeting cells that might escape the adaptive immune response triggered by the DC vaccine [6]. Adding a third component, nivolumab, an immune checkpoint inhibitor that targets the programmed cell death-1 (PD-1) pathway, further enhances the immune response [7]. Nivolumab works by blocking the inhibitory signals that cancer cells use to suppress immune activity, thereby preventing the 'turning off' of immune cells and enhancing the body’s ability to fight cancer [8]. This checkpoint blockade can potentially restore and amplify innate and adaptive immune responses, making it an ideal complement to DC and NK cell therapies [1]. This case report explores the synergistic potential of combining these therapies in the treatment of advanced hepatocellular carcinoma (HCC), focusing on the biological rationale and clinical outcomes of this multi-modal approach using DC vaccines targeting WT1 and α-Galcer, NK cell therapy, and Nivolumab.

## Case presentation

**Figure 1:**
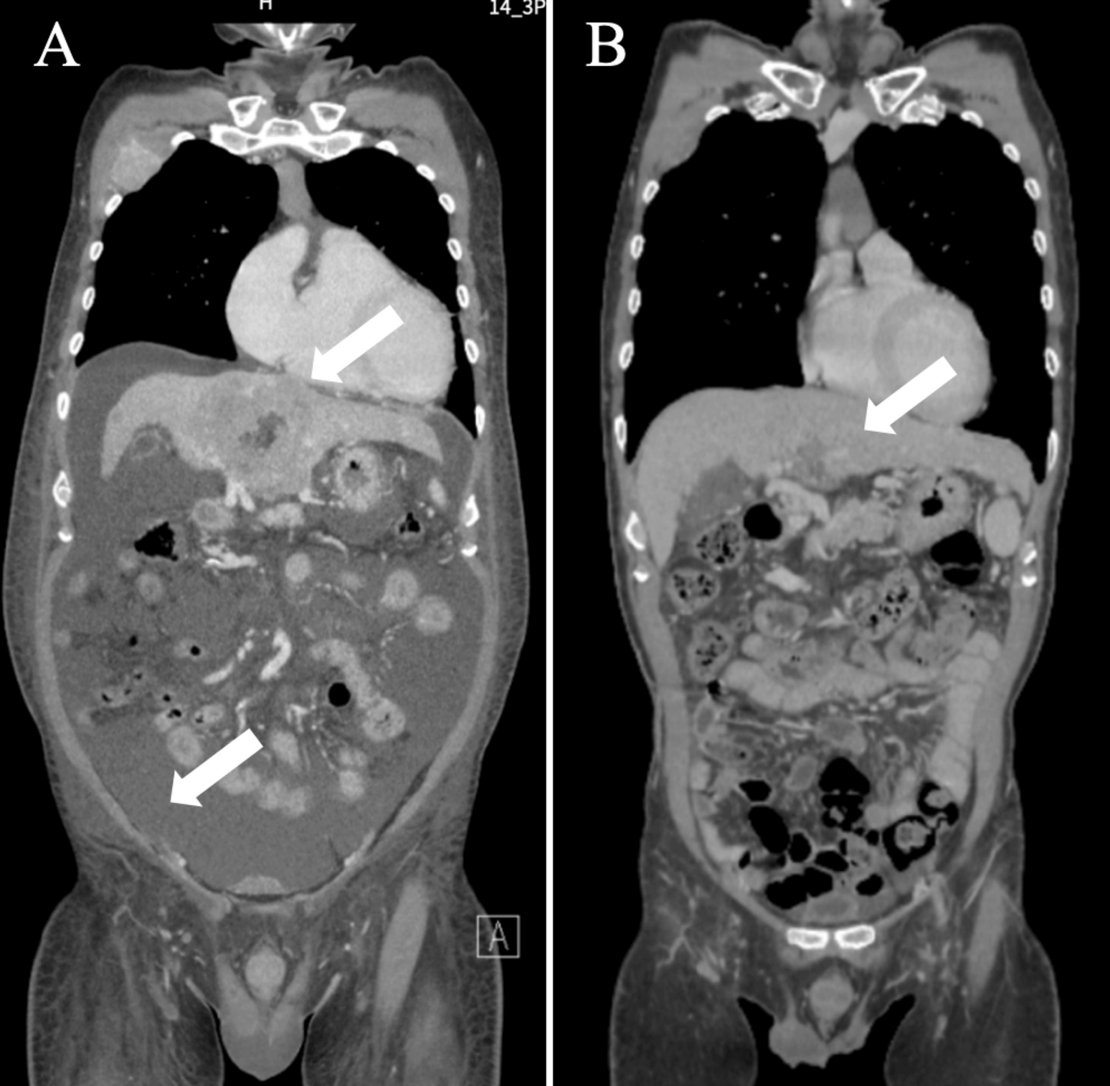
Initial status of the patients at administration, as shown by a CT scan of the coronal plane. CT: computed tomography.

**Figure 2:**
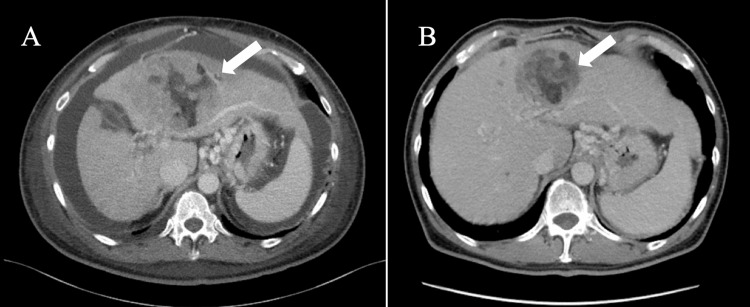
Improved condition after combined immune therapy, as the CT of the transverse plane shows. CT: computed tomography.

A 61-year-old man with a medical history of hepatitis C virus infection was admitted to the hospital. He presented to the outpatient department with abdominal distension and was admitted emergently due to abundant abdominal ascites of more than 3 liters. Upon examination at the facility, HCC T4N1M1 stage ⅣB (multiple lymph node metastases, multiple lung metastases, and bone metastases) was identified (Figures 1A, 2A). The patient was also in a pronounced state of ascites accumulation due to portal hypertension caused by the tumor. The laboratory findings showed he had a relatively low ratio of lymphocytes, impaired live function, and a high level of tumor markers (Table 1). Given the poor liver reserve, as indicated by a Child-Pugh classification of Grade C, pharmacotherapy proved challenging, leading to the adoption of a palliative care approach.

**Table 1:** Laboratory findings of the patients before and after the treatment. WBC: white blood cell; T-Bil: total bilirubin; AST: aspartate aminotransferase; ALT: alanine aminotransferase; CRP: C-reaction protein; PT: prothrombin time; PT-INR: prothrombin time-international normalized ratio; APTT: activated partial thromboplastin time; PIVKA-Ⅱ: protein induced by vitamin K absence-II; AFP: α‐fetoprotein; HBsAg: hepatitis B surface antigen; HCV: hepatitis C virus.

	Before the treatment	After the treatment	Reference
WBC	13,600	5,700	3,900～9,700/mL
Neutrophil	62.5	57.5	37%～72％
Lymphocytes	10.4	32.2	25%～48％
Platelets	10.0	6.6	15.3～34.6 ×10^4^//mL
T-Bil	3.2	1.5	0.4～1.2 mg/dL
AST	98	74	5～37 U/L
ALT	60	81	6～43 U/L
Albumin	2.3	3.0	4.0～5.2 g/dL
CRP	0.96	0.12	< 0.30 mg/dL
PT	48.2%	67.9	70～130%
PT-INR	1.63	1.30	0.85～1.15 s
APTT	29.6	31.2	24～34 s
PIVKA-Ⅱ	7283	19	< 40 mAU/mL
AFP	³70000	315.6	< 10 ng/mL
HBsAg	Negative	Negative	Negative
HCV antibody	Positive	Positive	Negative

One week after the diagnosis, the patient arrived at our clinic in a wheelchair. Given his poor performance status (PS) and the extensive volume of HCC, a relatively intensive immunotherapy plan was designed. Apheresis was conducted the following day, and two weeks later, the DC vaccine (targeting WT1 and α-Galcer) along with Nivolumab was administered weekly for the first four courses, then biweekly for a total of seven courses. Additionally, a combination of NK cell therapy was administered every three weeks, totaling three courses. After the first three courses of combination immune chemotherapy, the patient's PS improved, and his abdominal tension was significantly alleviated. At the end of the sixth course of combined immune therapy, the patient was referred to the previous hospital to reassess the liver reserve, which had improved to Grade A in the Child-Pugh classification. CT scans showed that the size of both primary tumors and metastases was reduced, and abdominal ascites had disappeared, indicating a partial response according to Response Evaluation Criteria in Solid Tumors (RECIST) criteria 1.1 (Figures 1B, 2B). Tumor markers also decreased significantly after the treatment, with the protein induced by vitamin K absence-II returning to a normal range. The general condition of white blood cells and the ratio of lymphocytes also improved to a normal range. Based on the improvement in liver reserve, standard chemotherapy with Atezolizumab plus Bevacizumab was initiated after the seventh course of combined immune therapy.

## Discussion

This case report illustrates a notable instance where immune combination therapy was highly effective for a patient with HCC. There are no effective treatment options for patients with poor PS and limited liver reserve, a particularly harsh reality for younger individuals [9]. In this case, the use of immune combination therapy not only enhanced anti-tumor effects but also improved PS and liver reserve. Standard treatments were previously considered unsuitable and are now possible. Immune combination therapy may be regarded as an essential option for patients unfit for standard therapies recommended by guidelines.

Traditional chemotherapy agents are recognized for selectively targeting and destroying rapidly proliferating cancer cells. However, it is increasingly understood that their effectiveness also stems from their capacity to boost anticancer immunity [10]. This occurs either through releasing immune-activating molecules from dying tumor cells or indirectly impacting other immune cells. DCs are harvested from the patient and modified ex vivo to promote an immune response aimed at tumor eradication. Numerous strategies are being tested to enhance long-term anti-tumor responses by DCs. Furthermore, combining DC vaccines with other treatments, such as chemotherapy and monoclonal antibodies, could lead to more effective cancer therapies [11]. NK cells are crucial for identifying and attacking various stressed cells, including tumor and virus-infected cells. NK cells perform direct attacks on these targets and contribute to orchestrating and sustaining broader immune responses [12]. Blocking immune checkpoint pathways, which cancer cells use to evade detection as standard body components, is a promising strategy for achieving anti-cancer immunity. Numerous agents are undergoing extensive clinical evaluation, as discussed in several review articles [13].

However, immune combination therapy has several limitations. First, its high cost has hindered widespread clinical usage. Second, the conditions for cell incubation vary with each sample in different cases, influenced by various factors related to the patient's condition. Third, there is no standardized protocol for the interval and dosage of incubated cells across different cancers, even within the same cancer type.

## Conclusions

An advanced HCC patient, initially deemed unsuitable for standard treatment, demonstrated remarkable improvement after receiving combined immune therapy, which was subsequently followed by standard treatment. This combined immune therapy represents a potentially significant treatment option for patients with advanced hepatocellular carcinoma and poor liver reserve function. It is particularly promising for relatively young patients, offering hope and improved outcomes where conventional treatments might not be viable.
